# A Critical and Anatomical Examination of the Parts Immediately Interested in the Operation for a Cataract; with an Attempt to Render the Operation Itself, Whether by Depression or Extraction, More Certain and Successful

**Published:** 1789

**Authors:** Silvester O'Halloran

**Affiliations:** Honorary Member of the Royal College of Surgeons in Ireland, and Surgeon to the County of Limerick Hospital


V. *A critical and anatomical Examination of the Parts immediately intereſted in the Operation for a Cataract; with an Attempt to render the Operation itſelf, whether by Depreſſion or Extraction, more certain and ſucceſsful. By* Silveſter O'Halloran, *Eſq. M. R. I. A. Honorary Member of the Royal College of Surgeons in Ireland, and Surgeon to the County of Limerick Hoſpital.*— From *The Tranſactions of the Royal Iriſh Academy*, 1788. 4to. Dublin.

---

*Nullius addictus jurare, in verba magiſtri.* Hor.

---

THOUGH it has been unanimouſly agreed on, by both ancients and moderns, that the cataract is an opaque body immediately behind the pupilla, oppoſing the paſſage of the rays of light to the bottom of the eye; and that the cure of this diſorder conſiſts in removing this opacity; yet the part immediately diſeaſed has been for about a century the ſubject of much controverſy, whilſt the operation itſelf, the moſt eſſential point of inquiry, ſeems as uncertain now as it was a thouſand years ago, notwithſtanding the boaſted improvements of M. Daviel, and other moderns.

The ancients ſuppoſed that the cryſtalline lens was the principal ſeat of viſion, which they agreed to place in the centre of the eye; that the ſpace between it and the bottom of the eye was filled by the vitreous humour, and that the aqueous humour occupied the anterior part of this organ. As the iris interſected this laſt ſpace, they agreed to call anterior, or outer chamber of the aqueous humour, the parts between it and the cornea tranſparens; and poſterior, or inner chamber, what remained between the cryſtalline lens and it. The cataract, it was affirmed, was a web or membrane formed immediately behind the pupilla, in this poſterior chamber, and far removed from the cryſtalline, not unlike a ſcum that is ſometimes found on the top of bottled liquors not well corked. But as experience proved that people, after the removal of this opaque body, by no means ſaw with that diſtinctneſs that might be expected, and that theory and practice might go hand in hand, this phænomenon was accounted for by obferving, " that in the formation of this fcum, " or membrane, the moft denfe parts of the " aqueous humour were engaged, the remain- " der of this liquor was therefore rarer, or lefs " enabled to caufe a convergence of the rays of " light, and fight muft of courfe be propor- " tionably weaker."

Towards the decline of the laft, but particularly fince the commencement of the prefent century, repeated diffections and obfervations made it but too evident that the cataract was not a membrane, but the cryftalline lens itfelf, that was rendered opaque. Numbers of cafes, and many works, were publifhed, from time to time, to corroborate this fact, which were violently oppofed by the partizans of the former doctrine: the chief of their arguments, and, for the time, the moft difficult to be anfwered, was this—" It is an acknowledged fact that the " cryftalline is placed in the centre of the eye; " but every oculift knows that the opaque " body to be removed lies immediately behind " the pupilla; therefore it muft be a mem- " brane between the cryftalline and iris."— This put the advocates for the new doctrine on on a closer examination of the structure of the eye, in which Brisseau, Maitre Jean and St. Yves, but more particularly Heister, Morgagni, Petit, and Winslow, bore distinguished parts. Every new inquiry contributed to advance the seat of the crystalline more forward, till at length, in the year 1729, Doctor Petit published a letter * in answer to some remarks of Hequet's, in which he demonstrated that the crystalline was so near the pupilla that it was impossible to introduce a cataract needle between it and the iris without wounding it: and to make clear to every conception this fact, he gave with this letter a figure of the eye, more correct than any that had yet appeared. Still he kept up the distinction of the different chambers of the eye, and in this figure determined their limits, which have been carefully noted by all subsequent writers. But that moderns should appear no more defective in point of theory and optics than the ancients, the limited sight that followed the operation was accounted for by observing, "that the crystalline "is a denser medium than either the aqueous "or vitreous humour, and of course, by its

* Sur la vrai situation du Cristalin.

" removal, ſight ſhould be proportionably

" weaker."

*Anatomical Obſervations on the Structure of the Iris, Situation of the Cryſtalline, &c.*

After names ſo reſpectable and truly great as thoſe of Heiſter, Petit, Morgagni, and Winſlow, one would naturally imagine that nothing with reſpect to the ſtructure of the eye was left unexplored; yet, from the following account, it will appear that much was ſtill wanting. I ſhall not enter into a general deſcription of the eye, but on the preſent occaſion confine my remarks to the parts immediately intereſted in the operation, and to the errors committed in the deſcription of them. And firſt; much confuſion has ariſen on account of the two chambers of the aqueous humour, and very reprehenſible miſtakes in the deſcription and delineation of the iris: the iris is generally ſuppoſed to take its riſe from the ſclerotica, at its junction with the cornea tranſparens; but though this is exactly the caſe at the middle of the ſuperior and inferior parts of the eye, as it lies in the orbit, yet the adheſion of the ligamentum ciliare gradually falls back on the ſclerotica as it advances towards the two canthuſes, inſomuch insomuch that here the origin of the iris is a mathematical line, posterior to that of the cornea transparens; a remark of great consequence to the operation, but particularly to the extraction of the crystalline. To prove this, if you remove the cornea transparens at its junction with the sclerotica, you will evidently see that the close adhesion between this last and the choroides, called ligamentum ciliare, is exactly as described. The uvea, or iris, is also represented as proceeding exactly flat, from the edge of the sclerotica to its aperture called the pupilla; yet if we look into an human eye, or into that of any animal, we shall clearly see that the iris, so far from being flat, is very convex, and that this convexity is greatest at its sides. If besides viewing closely the eyes of living animals, we examine through the cornea of inanimate ones, we shall perceive the same appearance. Certain it is, that after cutting off the cornea lucida, the situation in which the eye is placed being mostly on its posterior extremity, makes the whole eye, and of course the iris, appear flatter than they really are; but a little reflection, and an alteration in the position of the parts, will soon prove the fallacy of this appearance; for placing the sides of the eye nearly horizontal, horizontal, (according to their natural ſituation) you will quickly ſee the iris aſſume a much more convex appearance, provided in removing the cornea you have not injured the cryſtalline capſula, even though the loſs of this cornea ſhould have made the parts leſs compact.

Indeed, from the days of Galen, the convexity of the iris was never doubted, till Veſalius firſt pretended to controvert this truth; and all the figures given of it by former anatomiſts and opticians have ſo repreſented it, notwithſtanding that they agreed to place the cryſtalline in the center of the eye. But ſince Monſ. Petit (already quoted) has affirmed that the iris is flat, and as ſuch has repreſented it, he has been in this error followed by ſubſequent writers; yet that it is an error, and with reſpect to the operation of extraction a very alarming one, will appear from the following exact deſcription:

The vitreous humour occupies all the poſterior and anterior part of the eye as far as the iris, leaving a ſmall ſocket or cavity in its anterior part for the lodgement of the cryſtalline lens. It is ſaid to be covered by a fine membrane, called tunica vitrea; but for my own

 part, part, who have diffected as many eyes, and of different animals, as I believe any man, I confefs I have never been able to trace any membrane furrounding it except in its anterior part, and there it is covered very fenfibly and very remarkably. Leaving then the defcription of this tunica vitrea to thofe that can find it, I fhall obferve, that when the vitreous humour reaches the iris, there is a clofe adhefion between them by the intervention of a firm pellucid membrane arifing from the infide of the choroides, exactly oppofite to that part where the adherence between this laft and the fclerotica commences, called ligamentum ciliare. This membrane covers all the anterior part of the vitreous humour; but when it reaches the focket or cavity in which the cryftalline is contained it feparates; the pofterior, and by much the finer part, lines this focket, whilft its anterior one covers the cryftalline, fo that it becomes enclofed in it as a nut is in its fhell. Thus the cryftalline is enclofed in a fine pellucid membrane; which capfula is conftantly humected with a tranfparent liquor, which prevents any kind of adhefion or connexion between it and this interpofed body. The anterior part of this capfula is fo denfe as to be fometimes

ſometimes capable of being ſeparated into two diſtinct coats; the contained liquor is, from its diſcoverer, called Morgagni's liquor.

I have ſaid, contrary to all anatomiſts, that the inſide of the iris adheres cloſely to the anterior part of the vitreous humour, except where it opens for the lodgement of the cryſtalline; and the better to comprehend this fact, I ſhall give a new deſcription of the iris. With other anatomiſts, I always imagined that this laſt was a real continuation of the choroides; I am now ſatisfied that it is not, and that the aſſertion is very nearly as abſurd as to affirm that the diaphragm is a continuation of the pleura, though the choroides adheres pretty cloſely to the ſclerotica, near the inſertion of the optic nerve; yet from thence to the ligamentum ciliare the correſpondence is moſtly kept up by blood veſſels and nerves paſſing from one to the other. Here a cloſe adheſion of the choroides to the ſclerotica commences. At the middle of the ſuperior and inferior parts of the eye it begins at the very edge of the ſclerotica, bordering on the cornea tranſparens, but from thence to the two canthuſes it gradually retires back on the ſclerotica: the adhering part from the choroides, called ligamentum ciliare, is truly

 tendinous, tendinous, and forms an expanſion or covering to the iris; withinſide this are groupes of blood veſſels from the arterial circle of the iris, proceeding in nearly ſtraight lines as well to the pupilla as to the ciliary ligament. To prove that the iris is totally different from the choroides, and truly muſcular, it is only neceſſary to obſerve, that the inſide of the ligamentum ciliare, anſwering to its breadth, is fleſhy and thicker than any other part of this body; its fibres proceed radiated, or nearly ſo, from thence towards the iris. Here the covering of the anterior part of the vitreous membrane commences, and ſo cloſely is this attached to theſe radiated fibres, that their impreſſions are ſunk deep into it, and may be called the ſulci of the proceſſus ciliares. The firſt range of fibres on the inſide of the iris is, in a human eye, about the breadth of a line; a kind of tendinous narrow and circular band cloſes this phalanx, and from thence proceeds a ſecond row of radiated fibres thinner than the firſt; theſe alſo adhere and leave their impreſſions on the vitreous membrane; and that part of the iris which forms the pupilla is ſtill finer than the laſt mentioned, reſts on the cryſtalline, and is quite free from any adherence, by which means it contracts or dilates in proportion to the vicinity or diftance of objects. Thus the convexity of the iris follows nearly that of the cornea tranfparens, and is occafioned by the protuberance of the cryftalline; fo that the idea of a pofterior chamber of the aqueous humour muft be for ever banifhed: nor is that of circular fibres belonging to the iris better founded in truth and anatomy. Thefe laft we are conftantly told were formed for the purpofe of contracting, as the radial ones were for expanding, the pupilla; but not to advert to a fact, which is, that the ftate of quiefcence in the pupilla is its dilatability, which is evident, becaufe when afleep, or in a ftate of inattention with refpect to objects, we conftantly find it fo: I fhall juft obferve, that there are none but radial fibres through the whole internal furface of the iris. That the convexity of the iris may be proved beyond a poffibility of doubt, let the fide of the cornea be pierced at its junction with the fclerotica by a lancet or cataract needle, and paffed in that direction to the oppofite fide of the eye. On examination you will find, that, befides the cornea, you will have wounded the iris a line higher than the ligamentum ciliare. If you perforate another eye a line and an half higher up up on the cornea, it will juſt glide over the pupilla, and from this to the top of the cornea within is another line. If from the ſummit of the cornea a ſtraight line be drawn, and parallel to one from the riſe of the iris, *i. e.* the ligamentum ciliare at the ſides of the eye, the diſtance will be found to be three lines and an half. Thus the diſtance between the riſe of the iris and the pupilla, or its upper extremity, is generally two lines and an half, oftener more, meaſured from either canthus; but from the middle of the ſuperior and inferior parts of the eye, as it lies in the orbit, a line leſs.

*Idea of adherent Cataracts exploded; real Difficulties attendant on depreſſing Cataracts demonſtrated, with the moſt rational Means of overcoming them.*

Briſſeau, Maitre Jean, Heiſter, and, in ſhort, all oculiſts, whilſt, as anatomiſts, they inform us that the cryſtalline is ſurrounded by a fine pellucid membrane, as operators, they are careful to tell us that the cataract frequently adheres to different parts of the iris. Heiſter, though his treatiſe *De Cataracta* merits high applauſe, ſeems ſo perſuaded of this imaginary adheſion, that, in his ſurgery, he directs, rects, when it is found ſo ſtrong as not to be ſeparated by the needle, to perforate the centre of the cryſtalline, in hopes of giving ſome ſmall admiſſion to the rays of light. Warner, who we ſhould ſuppoſe always paid particular attention to this organ, though he tells us that the cryſtalline is inveſted by a fine membrane, from which it readily eſcapes by the leaſt aperture, yet attempts to determine, as an operator, whether there be an adheſion of the cataract to the iris or not *; nor can his method of performing the operation of depreſſion be approved of, ſeeing that he directs the needle to pierce the ſclerotica at a very ſmall diſtance from the cornea, by which means the iris muſt unavoidably be wounded. In a word, the adherence of cataracts has been the language of antiquity, and continues to be that of modern times; but it certainly is not the language of anatomy or reflection, for it is not the language of common ſenſe. But before we proceed to explain what has given riſe to this imaginary adheſion, the following practical remarks on the different humours appear very ſeaſonable:

* Deſcription of the Eye and its Diſorders, page 81, &c.

And

And firft, as to the aqueous humour, it is a fact long eftablifhed, that if, by a wound of the cornea, it efcapes, it becomes in a very fhort time replenifhed; and the procefs of extracting the cryftalline proves that this regenerated liquor is as well adapted to all the purpofes of vifion as the former. The vitreous humour, if partly or totally loft, never can be reftored; but a wound of this body does not deftroy its tranfparency, nor even injure it, as is demonftrable by the procefs of couching, which cannot be effected without not only wounding but feparating parts of it, and forcing the cryftalline through them. A wound of the cryftalline is conftantly followed by its opacity, as numbers of experiments prove, many of which are within my own knowledge; and a fevere compreffion of it will produce the fame effect. This lens, when fairly difcharged from its capfula, and lodged under the vitreous humour, infenfibly waftes away *; but I have had proofs that when it flips into the aqueous liquor this is by no means the cafe. A difeafed cryftalline, whether hard or foft, is conftantly found fmaller than a found one; and its capfula or cover-

* Philofophical Tranfactions for 1730, No. 384.

ing, ing, it may be affirmed, whilſt entire, is always tranſparent, let the ſtate of its interpoſed body be what it may. A wound of this membrane ſoon heals; and though by it Morgagni's liquor may eſcape, yet it alſo becomes ſoon recruited. Both theſe intereſting facts are proved by couching; for if you fail of depreſſing the cataract ever ſo often, yet you may at length ſucceed; and though you ſhould fail in this, yet you are certain to remove the opacity by extraction, which could never happen did not the different wounds of this capſula heal, and the incloſed liquor regenerate.

We will now ſuppoſe a perſon preſents himſelf for the operation; the cataract is of a pearl colour, greyiſh or white; the eye feels plump, the pupilla contracts and dilates, and the patient diſtinguiſhes light and darkneſs: a better-conditioned cataract cannot offer, nor a fairer for depreſſion. Let us now ſee what are the real, not imaginary, obſtacles to the ſucceſs of the operation: the needle pierces the ſclerotica, we behold it, through the pupilla, lodged in the cryſtalline; the ſurgeon endeavours to diſengage and remove the cataract; it ſeems, in part, obedient to the needle; as it is preſſed down, the iris ſeems to follow it, but lighten the force, and every part aſſumes its former place and appearance; you renew your endeavours, and on preſſing the cataract below the pupilla, and retaining it there awhile with the needle, the diaphanous vitreous humour follows it, and for the inſtant enables the patient to ſee objects; the needle is now carefully withdrawn, and all parties congratulated on the ſucceſs of the operation. It is, however, but tranſitory, for the parts return to their former ſituation, and any violence done to the vitreous membrane is removed before the eye is again opened. Let us ſuppoſe, in the firſt inſtance, that the operator ſees the cryſtalline riſing: perſuaded that this is occaſioned by its adherences, he freely pricks and wounds the proceſſus ciliares, which are the internal parts of the iris, to break this coheſion; the hæmorrhage diſturbs his plan by deſtroying the tranſparency of the aqueous humour, and he withdraws his needle *re infectâ*; or if he perſeveres, he may have the credit of deſtroying the eye in forming this ſeparation. Here are in one view collected all the proofs, and melancholy ones they are, of an adherent cataract; but the deſcription already given will clearly explain them. It is to be remembered, that the opaque cryſ-

talline talline has a lodgement formed for itſelf in the anterior part of the vitreous humour; that it is ſurrounded on every ſide by a ſtrong membrane, which is a continuation of that which covers the anterior part of this laſt body; that the proceſſus ciliares, being the inſide of the iris, adhere cloſely to this membrane in every part, even to the border or edge of the cryſtalline capſula, to which capſula the cataract has not the ſmalleſt adherence; the foſſula, or bed, alone muſt give ſome degree of ſtability to the cryſtalline; but when to this we add its envelope, the covering of the iris, and its ſtrong adheſion to the vitreous membrane, we muſt be convinced that nature has paid uncommon attention to the ſecurity of this body, and that no ſmall pains and attention are neceſſary to diſplace it. Certain it is, that if a ſufficient opening be made in the capſula, the cryſtalline may be thrown out of it by means of its contained liquor. But are the ſmall-pointed needles, moſtly uſed, well calculated for this purpoſe? They undoubtedly are not: they perforate this membrane, and ſtick in the cryſtalline, which is of a thickiſh viſcous ſubſtance, often much harder than in its natural ſtate. Pains are taken to remove this opaque body, but the

 needle needle does not afford a ſufficient paſſage for its exit; the parts are preſſed down, and the vitreous membrane, and of courſe the iris, muſt yield to this preſſure, from their connexions with each other, without the aid of any imaginary adherence of the cryſtalline or its capſula to the iris: but let us ſuppoſe the cataract fairly diſlodged from its bed by a proper opening of its capſula; are there no other obſtacles to its precipitation? There are, and conſiderable ones; the vitreous membrane and its adheſion to the iris oppoſe it; ſo does the denſity of the vitreous humour itſelf. Theſe are now the real difficulties, and none other. It is for theſe reaſons that the cataract ſometimes ſlips into the watery chamber of the eye, which, from its tenuity, gives leſs reſiſtance to it; and it is this circumſtance that gave riſe to Monſ. Daviel's method of extracting the cryſtalline.

To overcome theſe real obſtacles, care muſt be taken, firſt, by a proper opening of the cryſtalline capſula, to give room to the diſcharge of this body; next, to diſlodge it from its ſocket or bed; and laſtly, to withdraw it to the poſterior and inferior parts of the eye, at leaſt to place it below the pupilla. Inſtead of the needles in uſe, I have mine flat, pointed, and edged edged like lancets, and, like them, gradually increafing in furface. The length of the incifive part is to its greateft breadth nearly as three to two; from the broadeft part it rounds off gradually; and both the handle and blade are fhorter than thofe of the common cataract needle. With this knife or lancet, moiftened, the eye (if the left) is to be perforated in the fclerotica, at about two lines diftance from the cornea lucida, at the external canthus. If for the right eye, and the operator is not ambo-dexter, a curvature may be made in the inftrument, and the lancet fhould pierce the eye at the internal canthus, and at the fame diftance from the cornea tranfparens. Let it advance in nearly a ftraight line, (for it fhould have a fmall inclination towards the pupilla) and it will then enter into the fide of the capfula. The breadth of the inftrument alone will give a tolerable opening, which fhould be increafed by a gentle elevation and depreffion of the fides (not the point) of the inftrument. Pufh the point after this to the other fide of the capfula, which is alfo to be opened, but without injuring the proceffus ciliares. Sufficient fpace is now left for diflodging the cataract from its bed, which the furrounding fluid will

4 facilitate; facilitate; but if we fail in our endeavours the capſula will heal, and all will be to do over again. To effect this, the cataract ſhould be gradually diſengaged by gentle and nearly rotatory motions, and inſenſibly withdrawing it from before the pupilla, which the breadth of the needle ſeems well calculated for. When you behold it retiring from it, you ſhould gently preſs it (but with the ſides of the inſtrument) to the bottom of the eye, and there detain it for two or three ſeconds, or till the vitreous humour fills up the deſerted place. Though a cataract thus depreſſed may riſe a little, yet as it is effectually removed from its original place, it will inſenſibly fall again, and melt away; for I am to repeat it, that the ſucceſs of the operation depends on a ſufficient opening of the membrane, and puſhing the opaque body from its natural reſting place. If the cataract be in a diſſolved ſtate, the firſt perforation will give iſſue to it; and though it may appear to diſturb the other humours, yet in a few days all will inſenſibly ſubſide, and the eye clear up. There are other inſtances where the cryſtalline will be removed with very little trouble to the operator; but this only proves that its membrane is very thin, and the vitreous ous humour not fo vifcid as ufual. But notwithftanding the directions given to remove the cryftalline, if it fhould ftill refift our endeavours, and if, by accidentally wounding the iris, blood fhould follow, in this cafe, when the operator is fatisfied that the capfula is fufficiently dilated, he fhould immediately withdraw the needle, and prefs with his finger on the fide of the globe oppofite the perforation, and this will effectually diflodge the cataract, without any injury whatever to the eye, provided the preffure is not too violent, which the forcing out the cryftalline does not feem to demand.

Having, I think, effectually exploded the erroneous doctrine of adherent cataracts, and given a more exact defcription of the parts interefted in the operation, the true caufes of the difficulties that occur in it, and the means of overcoming them, than has hitherto appeared, I fhall now proceed to treat of the extraction of the cryftalline, and propofe an operation much more fimple than that now in ufe, and attended with infinitely greater advantages to the patient.

*Of extracting the Cryſtalline.*

Wounds of the cornea have been long known to be attended with no danger or inconveniency, except from the cicatrice obſtructing the rays of light; for the aqueous humour is ſoon reſtored. Pieces of the cryſtalline have been often known to paſs into this chamber, and to be ſometimes extracted by inciſing the cornea; inſtances of which are given by Mery, Petit, and St. Yves; and, encouraged by this ſucceſs, Mery propoſed to the Academy the extracting the cataract, by an inciſion of the cornea, as a certain cure *. It does not, however, appear that he ever reduced it to practice; and whatever applauſe is due to this method, M. Daviel is certainly entitled to it. He pierced the cornea nearly in a line with the pupilla, at the external canthus, with a cataract needle, and continued it in this direction till it paſſed through the oppoſite ſide of this coat. The ſide of a fine ſciſſars was introduced into the firſt aperture, and the inferior half of the cornea was divided near the ſclerotica; another needle opened the cryſtalline membrane; and by a

* Memoires de l'Academie des Sciences, 1707.

gentle gentle preſſure on the globe of the eye the cataract ſlipped into the aqueous chamber, and ſo down the cheek. Such, in a few words, is M. Daviel's account of this operation. Succeeding writers have laboured to reduce the operation to greater ſimplicity; for it was found that, beſides the cicatrice from the wound, the ſqueezing of the blades of the ſciſſars added conſiderably to the opacity. A ſimple inſtrument, ſomething like an iris knife, has been recommended, and is generally uſed, to perform the entire inciſion of the cornea with.

M. La Faye* directs the cornea to be pierced at about half a line from the ſclerotica, and to puſh it on in a ſtraight line till it paſſes through the oppoſite ſide, when by a ſingle inclination of the inſtrument the inferior half of the cornea is at once divided: nor need you fear, ſays he, to hurt the iris in traverſing the cornea, as it is plane or flat in its ſurface, as Dr. Petit demonſtrated in the Memoirs of the Academy of Sciences for 1728. Mr. Warner † would have the knife to be ſuddenly and reſolutely puſhed through the cornea, and paſſed in a ſtraight

* Mem. de l'Academie de Chirurgie, Tom. VI. page 304.

† Deſcription of the Eye, page 101.

line to the other fide. Such alfo are the directions given by Sharp*, Bertrandi †, and all fubfequent writers.

Never was operation received with greater applaufe, or more fpeedily and univerfally adopted for thirty years paft, than the prefent. The avidity with which it was embraced proves but too truly the difficulty and uncertainty of that by depreffion, and the utility and neceffity of the prefent memoir. For, notwithftanding all that has been faid in its favour, I am not afraid to affirm, that never was operation lefs entitled to public eftimation. This declaration is not the refult of theory and fpeculation, but of found practice. I have myfelf performed it both with the fciffars as Daviel recommends, and with a knife of my own invention, and have frequently feen it performed by others, and never with fuccefs adequate to expectations. For, in the firft inftance, the femifection of the cornea leaves a cicatrice, by which nearly half of it becomes opaque, at leaft the rays of light cannot diftinctly pafs through it. But befides this defect, unavoida-

* Philofophical Tranfactions for 1753.

† Traité des Operations de Chirurgie, page 345.

ble ble by the directions given, there are other ſtill more alarming accidents to be apprehended from the very manner of piercing the cornea. We ſee La Faye recommends the needle to proceed in a ſtraight line from one ſide to the other, without fear of wounding the iris, which he tells you is flat. Warner deſires it to be paſſed ſuddenly and reſolutely; and ſuch is too much the practice. What are the conſequences of this rule? That the iris muſt infallibly be wounded; and this accounts for the complaints on all ſides, that a part, ſometimes the whole, of the vitreous humour is diſcharged with the cryſtalline. Examine the projection of the iris and cryſtalline, and you muſt ſubſcribe to this melancholy truth: but that no doubt ſhould remain, let it be remembered, that if the iris be not wounded, no particle of the vitreous humour can eſcape by moderately preſſing on the globe after opening the cornea. We have already noted that the anterior part of this humour is covered by a ſtrong membrane, firmly adhering to the proceſſus ciliares, except where it forms a ſheath for the cryſtalline; what then can paſs through the pupilla by preſſure but this cryſtalline? The vitreous membrane, and the adheſion of the iris to it, oppofe any other, except the preffure be too ftrong; but even in this cafe the cataract muft pafs through firft. If then a part of the vitreous humour efcape, and a diftorted iris follow, we muft attribute both to an abfolute mifconception of the ftructure of the parts, and to erroneous rules, the confequence of it. Thus we fee, befides the unavoidable cicatrice of the cornea, other and more alarming dangers are to be feared, even to the total lofs of fight, notwithftanding the cataract is removed.

But may not the ftructure of the parts furnifh fome hints to render this operation more fafe and certain? From a careful perufal of the foregoing very accurate defcription of them, I think it will; and the following is the modus I would recommend: — My knife is of the fame fize and figure of thofe ufed in this operation, except that it cuts at both fides from its point, and that the incifive parts are a little convex, the concave or infide of which fhould be marked in the handle. With the concave part next me I pierce the fclerotica, very near the edge of the cornea, fuppofe the third of a line, at either the external or internal canthus, according to the eye to be operated on. Inftead of pufhing it on in a ftraight line, as recommended, I direct the point rather a little towards the aqueous chamber than the iris, for fear of wounding this laſt, which its riſing convexity expoſes it to. The paſſage of the needle is proved by part of the aqueous humour's eſcape, and by your ſeeing its point, within the cornea, between it and the iris. You now inciſe the inferior ſide of the ſclerotica, advancing the inciſion to the edge of the cornea tranſparens, as the adherence between the iris and ſclerotica approaches cloſer to the cornea the farther you go from the ſides of the eye. Without withdrawing the inſtrument you cut the upper ſide of the ſclerotica in the ſame manner. The reaſon why the inferior inciſion is firſt performed is, that if you cut the upper ſide firſt, a little blood might oppoſe your carrying this inferior opening ſo very accurately afterwards. Thus nearly one ſide of the ſclerotica, from top to bottom, at its junction with the cornea, becomes divided: with the point of this very inſtrument you prick the cryſtalline capſula, and the ſmalleſt inclination of it inſide the pupilla will do this, and then gently preſs on the globe of the eye, the cataract will inſtantly ſlip out, and though divided into parts, as as it is ſometimes, will with the greateſt facility be extracted through the aperture.

By this ſimple mode of operating, very little, if any, opacity can appear on the cornea ; and though ſome may, yet, as it is at its very edge, from whence the rays of light ſeldom are tranſmitted to the bottom of the eye, no defect can follow ; whereas by inciſing half the cornea from ſide to ſide, and not confining the opening to its extremity, a very great opacity remains. I have made no allowance for the eſcape of the vitreous humour, becauſe it cannot poſſibly happen except the iris is wounded, and a very little attention will ever prevent that.

